# Assessing the Impact of a Community-Based Health and Nutrition Education on the Management of Diarrhea in an Urban District, Cairo, Egypt

**DOI:** 10.5539/gjhs.v8n2p46

**Published:** 2015-06-05

**Authors:** Shaimaa B. Abdel-Aziz, Maha A. Mowafy, Yasmine S. Galal

**Affiliations:** 1 Department of Public Health and Family Medicine, Cairo University, Cairo, Egypt

**Keywords:** diarrheal disease, children, health education intervention, health services

## Abstract

Diarrhea is considered as a major cause of mortality in children aged less than five years old. This pre/post interventional study was designed to assess maternal knowledge about diarrhea and implement a community-based health and nutrition education messages. The study was held in Al-Darb Al-Ahamar (ADAA) district, Cairo, Egypt and targeted a random sample of 600 mothers having at least one child under-five years old and complained of at least one previous attack of diarrhea. The study was conducted in three phases. The pre-intervention phase included a base line survey for the mothers and training activities for the community health workers (CHWs). Intervention phase included health and nutrition education sessions; performance evaluation for the CHWs during providing the message. In phase three, the mothers had no instructions for 3 months then the post- intervention interview and feedback sessions were conducted. Results showed that knowledge of mothers about diarrhea (etiological factors and preventive measures) had improved significantly after the intervention. During observation CHWs’ scored 50% of the required tasks in education and communication skills. In the feedback sessions, all the mothers declared that nutrition education sessions were highly valuable, and asked for on-going support and training programs. The current study found that health and nutrition education sessions were successful in improving mothers’ knowledge regarding preventive measures and management of diarrhea. CHWs are effective health education providers especially in household based intervention. Thus, health services should support community based interventions to reinforce mothers’ knowledge and practices towards their sick children.

## 1. Introduction

Diarrheal disease is an important public health problem among under-five children in developing countries (Kumar & Subita, 2012). Diarrheal diseases remained to be the second leading cause of death in children under-five years old, accounting for about 760,000 children deaths every year (World Health Organization [WHO], 2013). Different literatures show that the magnitude of childhood diarrheal disease varies across the globe from 3.6% in India, 21.4% in Iraq and 19.6% in Egypt to 32.7% in Tanzania (Kumar & Subita, 2012).

Studies have shown that, in developing countries like Egypt, the etiology of diarrheal disease among under-five children is complex and the relative contribution of each factor varies as a function of interaction between socioeconomic, environmental, and behavioral variables (Walker et al., 2007).

Since 1990s, the Egyptian National Control of Diarrheal Disease Program (NCDDP) aimed at improving case management through assured production, distribution and promotion of oral rehydration solution (ORS) use through mass media (Miller & Hirschhorn, 1995). However, the magnitude of improvement has been questioned by other studies reporting that diarrhea is still the leading cause of death in spite of substantial decline of diarrhea-related mortality (Egyptian Demographic and Health Survey [EDHS], 2014; Yassin, 2000). The EDHS 2014 reported that 14% of the children had diarrhea in the two weeks preceding the survey at the national level.

Diarrheal diseases still continue to be an important cause of morbidity and mortality worldwide in spite of all advances in health technology, improved management, and increased use of oral rehydration therapy in the past decades. Morbidity due to diarrhea has not shown a parallel decline in comparison to mortality trends, and global estimates remain between two and three episodes of diarrhea per under-five year child per year (WHO, 2013). Millennium Development Goal - 4 aimed to reduce childhood mortality by 2/3 by the year 2015, however previous studies (Alkizim, Matheka, & Murithi, 2011; EDHS, 2014) have showed minimal progress in this regard. Therefore, efforts must be made to review existing strategies and formulate newer ones.

Reduction of diarrhea-related morbidity depends on identifying effective interventions and delivering them to the population at risk. Identification of children at higher risk for diarrhea is a crucial step for a better and more efficient utilization of resources that are usually too limited in developing countries (Yassin, 2000). Knowledge, attitudes, and practice (KAP) studies that determine the knowledge of communities regarding diarrhea uncovered marked deficiencies in the level of education given to people at risk of contracting the disease (WHO, 2001 & 2013).

Although it is known that knowledge, attitudes and practices (KAPs) of mothers play a crucial role in terms of childhood diarrhea, community-based interventions to improve these aspects are few especially in developing countries (Boschi-Pinto, Velebit, & Shibuya, 2008).

The current challenges of child survival are therefore to improve access to basic knowledge and appropriate quality services for those who are at risk (WHO, 2004). This could not be achieved without exploring the existing level of knowledge and practices of mothers regarding child health with special emphasis on the effect of appropriate nutrition in preventing complications of diarrhea and the leading role of mothers in first line management of diarrheal children under-five years. The objectives of this study were to assess the maternal knowledge about diarrhea and implement health and nutrition education messages using a community-based approach.

## 2. Methodology

### 2.1 Design and Setting

This is a community-based health and nutrition-education intervention study with pre/post assessment. The study was conducted in Al-Darb Al-Ahamar (ADAA) district, Cairo, Egypt as a part of an Integrated Community Development Initiative (ICDI) project. The overall goal of the project in ADAA was to improve the knowledge and attitude of the community and raise public health awareness towards some health issues; diarrhea, malnutrition, anemia, smoking and addiction (United Nations [UN] - Habitat, 2008). The project was executed by a Unified Group (UG) Organization that focuses on improving child health and nutrition as it works through community development associations in Egypt to train physicians and CHWs as health and nutrition educators. The health and nutrition education components of the ICDI project were implemented in collaboration with the Community Medicine department, Faculty of Medicine, Cairo University.

Al Darb al-Ahmar is a densely populated district in the heart of historic Cairo. The region has an estimated area of 51,000 Km2. It is located in south of Al-Azhar mosque and the Khan al-Khalili bazaar. Despite its valuable cultural assets, ADAA is one of the most distressed neighborhoods. Over the past decades, the area has suffered from social, economic, cultural and environmental deterioration (Aga Khan Trust for Culture, 2005a).

The WHO definition for diarrhea which is the passage of three or more loose or watery stool was used to identify the target population, while the degree of dehydration was evaluated against the WHO criteria including the general condition, sunken eyes, degree of thirst, and degree of loss of skin elasticity (WHO, 2014).

Health and nutrition education messages were provided by 16 female CHWs selected from the same area. They were members of the Unified Group Organization working in technical and vocational fields in Al-Darb Al-Ahmar Housing Rehabilitation Programme (ADAAHRP) (Aga Khan Trust for Culture, 2005b), with secondary level of education. The field study of the current research started on October 1^st^ 2013 till June 30^th^ 2014.

### 2.2 Sampling and Data Collection

The total population in Al Darb AI-Ahmar district is around 100,000, 70% of which is below the poverty threshold (United Nations Children’s Fund [UNICEF], 2014). The total number of families is 10,830, living in 13 geographically distributed clusters. The target group of this study was mothers who had at least one child less than five years old and previously exposed to diarrhea, representing 35% of the total population in the district (Central Agency for Public Mobilization and Statistics [CAPMAS], 2013).

The sampling design included initially a non-probability purposive selection of the targeted district followed by employing a multi-stage sampling method to select a random sample out of the listed population clusters. First 8 clusters were selected randomly out of the 13 clusters, and then through proportional allocation, a systematic random sampling technique was employed to reach the households (every fourth household), which had at least one under- five child, who complained previously of at least one attack of diarrhea. A total of 600 mothers having children aged under-five years old were enrolled for the study and their houses were marked with an identification sign.

### 2.3 Measurement Tools

The work was implemented in three phases. Phase one: a baseline survey was conducted, lasted for three months, with the aim of measuring mothers’ knowledge in managing diarrhea at home. The questionnaire for mothers was designed by the researchers out of the existing literature (WHO, 2006; King, Glass, Bresee, & Duggan, 2003) and was composed of two parts: demographic characteristics and the section dedicated to assess mothers’ knowledge regarding diarrhea. There were 12 questions (types of questions, close-ended, with options, yes, no, do not know, how many for etiology, symptoms etc.) for assessment of mothers’ knowledge regarding: definition, symptoms, complications and treatment of diarrhea, feeding pattern of children during diarrheal attacks and 8 questions to assess mothers’ knowledge as regards etiological factors and preventive measures of diarrhea.

Training and evaluation of the CHWs took place during this phase also. A health and nutrition education package on the home management of diarrhea was formulated and tested based on the definition and principles of health education and the definition, etiology, assessment of complications, and management of diarrhea (WHO, 2006). This package was given in a five-day course with 35 working hours. The methods used in training were lectures, group discussion, a video show and role plays. CHWs were assessed during providing the education messages by means of 10 multiple choice questions using a pre/post training test and an observation check list to evaluate their median performance achieved score using the inter-quartile cutoff levels.

Phase two: the mothers were divided into 16 groups, two groups in each cluster, with almost 35-40 mothers in each group. Each group was supervised by one CHW. They conducted health and nutrition education sessions with the objective of sensitizing mothers about the necessity of oral rehydration during diarrhea, making them understand the importance of continuing breastfeeding during diarrhea, empowering them with knowledge about the importance, preparation, and use of ORS, making them aware of the need of daily preparation of ORS and the quantity required as per age of the child. The tools used in education were lectures, data show and flip charts.

Each CHW did one session every two weeks for three months, meeting in a different home for each session; total number of sessions was 96 sessions throughout the study period. The researchers made two visits to the designated households, and held a session once every month at the health center with the CHWs to consolidate the issues taught in the education message, followed by a meeting to discuss the different reports presented by the CHWs on the progress of work, and to resolve any issues or problems.

Phase three: the women had no instructions for three months and then the post-intervention interview was conducted using the same pre-intervention questionnaire. Two feedback sessions were organized with the CHWs and researchers with the objective of consolidating the concepts taught to the mothers in the intervention phase. These two sessions were attended by 74 mothers, 37 mothers in each session, some of them agreed to participate in the future planned health and nutrition education community project in ADAA, with a response rate of 12.5%. Peer learning was encouraged during the sessions, whereas, active participant mothers were encouraged to explain the concepts taught to their group in presence of the researchers. Communicating with a fellow mother helped the remaining mothers in the group to express themselves and share their views without hesitation. Moreover, this approach helped encourage more participation and held the mother’s interest in the feedback sessions.

### 2.4 Data Management and Statistical Analysis

Scoring of the participants’ responses was carried out, right answer received a score of one, while wrong or don’t know received nil. The total score for the mothers’ questionnaire was 20; total score for the CHWs’ questionnaire was 10 points before and after training. Performance scoring system of the observation check list was planned to assign (0) for bad and (1) for good.

Data were entered and analyzed using SPSS (Statistical Package for Social Science IBM version 21). For quantitative variables, mean, median and standard deviation were used for reporting, while frequencies and percentage were used for categorical variables. The pre/post-intervention phases were compared using Chi-square test and paired t-test for qualitative and quantitative data respectively. P value less than 0.05 was considered statistically significant.

### 2.5 Ethical Consideration

Ethical approval was obtained from the Ethical Committee at the Faculty of Medicine, Cairo University. After submitting an official letter to the district, permission was gained from the district directors. Before data collection, a written consent was obtained from each study participant after explanation of the study objectives. For those not willing to participate in the study, their right was respected to withdraw from the study at any time. Confidentiality and privacy were also assured.

## 3. Results

The mean age of mothers was 33.8 ± 4.3SD (with the minimum age of 18 and maximum of 43 years). Families with one child less than five years were 35.5%, with two children were 47.3%, three children were 11.8%, and only 5.4% had four children. In terms of employment, 80.4% of the participants were housewives and 19.6% were employed. The health center, educational programs and the physicians were the main sources of previous knowledge about diarrheal treatments (43.7%). Among the 600 mothers interviewed, 396 participants (66%) mentioned that the onset of diarrhea was in crawling babies and the rest mentioned that it was after two years of age.

Mothers’ knowledge as regard; definition of diarrhea, its symptoms, amount of food given during diarrhea, importance of fluid during the attack and knowledge about signs of dehydration had improved significantly after the intervention (P <0.001) (Table 1). Also the table summarizes information regarding awareness of mothers about ORS. In the pre- intervention phase, 68.2% of mothers had heard about ORS, whereas in the post intervention phase, awareness about ORS increased significantly (P<0.001) to reach 92.7%. About 24.7% & 29.7% of mothers in the pre-intervention phase knew the correct method of reconstitution of ORS from the packets locally available and the preparation of the alternate sugar salt solution at home, respectively. These figures increased significantly (P<0.001) reaching 97% and 83% respectively after the intervention. Pre-intervention, 44.3% of mothers knew that ORS should be prepared fresh daily. In the post-intervention period, there was a further significant increase in the proportion of mothers who were aware of this (99.7%, P<0.00l). Age-specific feeding of ORS was not considered relevant by most of the mothers (82.3%) before the intervention. However, the intervention brought sensitization about the importance of feeding ORS in correct amount, mothers who felt the importance of feeding ORS in age-specific doses, rose significantly (P<0.001) to 90.3% after the intervention. Awareness that breastfeeding should be continued during diarrhea increased significantly from 65.2% of the mothers to 96.7% after the intervention.

**Table 1:** Mothers’ knowledge about diarrhea pre/post-intervention, Al-Darb Al-Ahamar, Egypt

Variables	Pre-test		Post-test		p-value

	No. (n=600)	Percent (100%)	No. (n=600)	Percent (100%)	
What is meant by diarrhea	492	82.0	580	96.7	0.001
Knowing symptoms of the diarrhea	406	67.7	560	93.3	0.001
Aware that breast feeding should be continued during diarrhea	391	65.2	580	96.7	0.001
Aware that amount of fluid must be increased during diarrhea	420	70.0	573	95.5	0.001
Aware that amount of food should not be restricted or diluted during diarrhea	223	37.2	541	90.2	0.001
Knowing that dehydration is the most important complication of diarrhea	274	45.7	551	91.8	0.001
Knowing signs of dehydration	112	18.7	597	90.7	0.001
Know the term ORS*	409	68.2	556	92.7	0.001
Knowing correct method for reconstitution of ORS from the packets locally available	148	24.7	582	97.0	0.001
Knowing correct preparation of sugar-salt solution at home	178	29.7	498	83.0	0.001
Aware that ORS has to be prepared fresh everyday	266	44.3	598	99.7	0.001
ORS has to be fed in specific amount as per child’s age	106	17.7	542	90.3	0.001

ORS

* =Oral rehydration solution.

The mothers’ knowledge as regards etiological factors of diarrhea and its prevention and awareness that water should be colorless, odorless and tasteless, had improved significantly (P value <0.001) after the intervention (Table 2).

**Table 2:** Mothers’ knowledge as regard etiological factors and preventive measures of diarrhea, pre/post-intervention, Al-Darb Al-Ahamar, Egypt

Variables	Pre-test		Post-test		p-value

	No. (n=600)	Percent (100%)	No. (n=600)	Percent (100%)	
Etiological factors of diarrhea:					
Un clean hands	410	68.3	597	99.5	0.001
Unhealthy water	426	71.0	596	99.3	0.001
Contaminated food	426	71.0	368	61.3	0.001
Cold cooked food	88	14.7	553	92.2	0.001
Hand washing prevents diarrhea	455	75.8	599	99.8	0.001
Eat vegetables and fruits after washing and peeling	154	25.7	498	83.0	0.001
Food must be covered if left outside the refrigerator	454	75.7	597	99.5	0.001
Water had to be colorless, odorless and tasteless	474	79.0	578	96.3	0.001

The total knowledge score with reference to prevention and home management of diarrhea increased significantly (P<0.001) after health education (Table 3).

**Table 3:** Mothers’ total knowledge score pre/post-intervention, Al-Darb Al-Ahamar, Egypt

Variables	Pre-test (mean±SD)	Pre-test (mean±SD)	P-value
Knowledge about diarrhea	8.4± 2.8	11.7± 0.5	0.001
Knowledge about etiological factors and preventive measures of diarrhea	5.32± 2.52	7.26± 0.74	0.001
Total knowledge score	13.8± 3.9	19.1± 0.9	0.001

Statistical evaluation regarding CHWs’ knowledge revealed no significant difference before and after training, expect for their awareness as regard; principles of health education message and signs of dehydration, significant improvement had been noted after training (P<0.001) (Table 4).

**Table 4:** CHWs’ knowledge in the pre/post-training sessions, Al-Darb Al-Ahamar, Cairo, Egypt

Variables	Pre-test		Post-test		p-value

	No. (n=600)	Percent (100%)	No. (n=600)	Percent (100%)	
What is meant by health education	10	62.5	14	87.5	0.219
Principles of the health education message	7	43.8	15	93.8	0.008
Symptoms of diarrhea	10	62.5	14	87.5	0.219
Type of food given during diarrhea	12	75.0	15	93.8	0.375
Breast feeding during diarrhea	14	87.5	15	93.8	1.000
Signs of dehydration	8	50.0	15	93.8	0.039
Correct preparation of ORS	8	50.0	14	87.5	0.07
Correct preparation of sugar-salt solution at home	6	37.7	9	56.3	0.453
Eat vegetables and fruits after washing and peeling	15	93.8	15	93.8	1.000
Complications indicate hospital admission	10	56.3	14	87.5	0.219

The performance of the CHWs while providing the health and nutrition education message was illustrated in Table 5. The percent achieved score during observation was highest for their knowledge about the topics: using clear language and dedication to time, but lowest for their level of explanation and communication skills. Accordingly, CHWs who should fulfilled 11 items (100%) during observation only scored 50% of the required tasks in health education and communication skills by using the median inter-quartiles cutoff levels.

**Table 5:** Community health workers’ observation performance score, Al-Darb Al-Ahamar, Egypt

Demonstrated knowledge and skills performance score	CHWs (n=16)	Achieved score%
Good model for healthy behavior	8	50.0
Influential personality	7	43.8
Dedicated to time	9	56.3
Knowledge about the topic	9	56.3
Clear and understood language	9	56.3
Loudness of voice	8	50.0
Level of explanation	6	37.5
Communication skills	6	37.5
Be motive and believable	8	50.0
Active participation with the mothers	7	43.8
Give enough time for mother to speak and ask	6	37.5

Analysis of the feedback sessions for the mothers in the post-intervention phase revealed that all the mothers found that nutrition sessions were highly valuable from the medical aspect. However, they criticized the CHWs for not giving enough time for them to ask. Therefore, findings derived from performance evaluation in the sessions reflected that CHWs focus on medical knowledge rather than establishing educational dialogue with the mothers and that shortage in communication skills had negative impact on their performance. By asking the mothers about reflected change in their attitude, all of them highlighted the importance of hand washing with soap before and after eating; washing vegetables and fruits before eating to guard against diarrhea and; avoidance of eating cold cooked foods. Mothers demanded regularity of health and nutrition education messages to reinforce their learning knowledge and change in attitude. However, they mentioned that they were interested in hearing rather than receiving printed educational material. They also asked for on-going support and training through conducting nutrition training programs and some of them (12.5%) agreed on participating in future community-based educational project in Al Darb Al-Ahmar, Cairo, Egypt.

## 4. Discussion

The current study was conducted to assess maternal knowledge about diarrhea and implement health and nutrition education messages, using a community-based approach. Community based integrated approach is one of the most important strategies to improve child survival. Studies from developing countries have proved that educating mothers, the first care provider to the child, is essential to reduce child mortality (WHO, 2004).

In this study the respondents were mothers having children under-five years old. Significant improvement in mothers’ knowledge after the intervention as regards diarrhea and its management was consistent with the findings of other studies (Haroun, Mahfouz, Mukhtar, & Salah, 2010; Karamagi, Lubanga, Kiguli, Ekwaru, & Heggenhougen, 2004), proving that health education using CHWs is an effective means of improving mothers’ competence in caring for their children at home during episodes of diarrhea.

The study revealed that, in the post-intervention period, there was a significant (P<0.001) improvement in the acquaintance of mothers with the term ORS (92.7% vs. 68.2%); correct method of reconstitution of ORS locally available (97% vs. 24.7%) and preparation of sugar salt solution (83% vs. 29.7%); knowledge about daily fresh preparation of ORS (99.7% vs. 44.3%); and the importance of continuing breastfeeding during diarrhea (96.7% vs. 65.2%). Sensitization about age-specific feeding of ORS also improved significantly (P<0.001) from 17.7% to 90.3%. These findings were consistent with other studies done in India and Tehran (Kolahi & Shekarriz, 2008; Pahwa, Kumar & Toteja, 2010).

Another similar study showed that 18% of the studied mothers had adequate awareness about ORS and 17% knew the ingredients of the solution, however, following the training programs; 80% of them reached adequate knowledge and their awareness increased significantly (Pahwa et al., 2010). In another study conducted by Kolahi and Shekarriz (2008), it has been emphasized that educational intervention has had a positive impact on maternal attitude and practice. Similarly, other studies revealed that educational interventions could improve the mothers’ knowledge and practices in management of diarrhea (Haroun et al., 2010; Pahwa et al., 2010; Kolahi & Shekarriz, 2008).

Another comprehensive study conducted in India showed that 63% of Indian mothers were aware of ORS, whereas, only 27% of them took advantage of it for their children (Rishi, Bodakhe, & Tailang, 2003). Moreover, in a study conducted by Shah et al. (2012b), results revealed that despite three-fourths of women knew about ORS, only one-fourth used it when their children suffered from diarrhea. These studies suggest a discrepancy between the cognition of ORS and its utilization. Thus, a superficial understanding of ORS is not enough and it consistent efforts are required to emphasize the importance of ORS in resolving dehydration during diarrhea, particularly in children; since this group of age is more likely to be dehydrated quickly (Pahwa et al., 2010). Oral rehydration along with appropriate feeding in children with diarrhea can markedly decrease the morbidity and mortality by sixty percent (Adimora, Ikefuna, & Ilechukwu, 2011).

Dehydration is one of the important complications of diarrhea. In the current study, 45.7% of mothers considered dehydration as the most important complication following diarrhea. In another study conducted in Iran, similar results were achieved (Khalili, Mirshahi, Zarghami, Rajabnia, & Farahmand, 2013).

In contrast, low level of knowledge regarding complications of diarrhea had been noted in other studies (Othero, Orago, Groenewegen, Kaseje, & Otengah, 2008; Shah et al., 2012a). In Indonesia, only 38% of the mothers identified two or more precise signs of dehydration (MacDonald, Moralejo, & Matthews, 2007).

Although rehydration therapy is a key factor to reduce diarrhea-related mortality, appropriate feeding during and after convalescence is important to reduce malnutrition and its long term effects. However, across developing countries less than 25% of children with diarrhea consume appropriate food during illness (Van der Hoek, Feenstra, & Konradsen, 2002). In the present study, 65.2% of mothers’ maintained consistent breastfeeding during diarrhea and 70% knew the importance of increasing fluid intake during the attack. However, Kolahi and Shekarriz (2008) found that only 11% of mothers had increased the amount of breast milk or food in their children’s diet and 60% had discontinued both. The results of a study done in Norway emphasized the role of breastfeeding as the most important factor affecting the duration of diarrhea (Shah et al., 2012b).

Regarding the amount of food given to children during diarrhea, 62.8% of mothers in the current study thought that food should be restricted or diluted to be more tolerable by the gastrointestinal system. Those results may be attributed to the effect of media campaigns that focus on increasing fluids not food intake during diarrheal attacks. Similarly, another study showed that mothers are likely to offer more fluid than to give more food to sick child during diarrhea (Mohsin, Raza, & Ahmad, 2009). Therefore, Continuous Behavioral Change Communication (BCC) strategy is required to bring a significant change in attitude and practices.

In the present study, only 14.7% of mothers in the pre-intervention phase stated that diarrhea might occur because of eating cold cooked food and 25.7% knew that eating vegetables and fruits after washing and peeling will prevent diarrhea. However, other studies (Joseph & Naregal, 2014; Shah et al., 2012b) revealed that a higher percent of the mothers were aware that cold cooked food is one of the etiologic agents of diarrhea and it can be prevented by washing and peeling vegetables and fruits before eating. That difference could be attributed to the credibility of these issues in the other countries.

In the current study, the mean total knowledge score of mothers was 13.8 ± 3.9 SD, which increased in the post- test to 19.1 ± 0.9 SD. The above findings were supported by a similar study conducted in Al Maki area, Gezira state, which revealed that health education caused a significant improvement in mothers’ knowledge and practice with respect to homecare of under-five children with diarrhea (Haroun et al., 2010).

CHWs provide a critical and essential link with health systems and are a powerful force for promoting healthy behaviors. Several important studies highlight the importance of home visitation by CHWs to promote hand washing and its impact on childhood diarrhea. Current estimates state that practicing hand washing with soap at the proper times could reduce children deaths by one million (Greenland, Cairncross, & Curtis, 2012). A randomized controlled trial in which CHWs made routine weekly visits to all households to promote hand washing in an urban slum population in Karachi, Pakistan led to a 53% reduction in the incidence of childhood diarrhea (Luby et al., 2005).

Based on results of the present study, although there were no significant difference in CHWs’ knowledge before and after training for most of the studied items, yet their median percent achieved performance score (for the required tasks in health education and communication during observation) was unsatisfactory, accounting for only 50%. In another study (Pahwa et al., 2010), similar result was estimated, ensuring that CHWs need more training and supervision. This reflects some of the problems mentioned by the interviewed women in the feedback sessions, they said that sometimes the nutrition educator spoke quickly leaving no time for them to speak or ask.

In spite that diarrhea seems sometimes as an old and non-significant problem, but the reality is that diarrhea is still a very serious problem in developing countries (Motlagh, Heidarzadeh, Hashernian, & Dosstdar, 2012).

### 4.1 Conclusion and Recommendation

Findings of the present study showed that mothers’ knowledge about diarrhea had improved significantly after the intervention, which makes it clear that education in this population needs to be intensified. This could be achieved through repeated and focused health and nutrition education messages through CHWs that will consequently enhance maternal knowledge, have a positive effect on treatment of diarrhea and improve health status of their children in the community. Finally, we recommend improving different approaches in health education to be an effective strategy for improvement of mothers’ competence in managing their children at home. Providing information through mass media and clarifying public information about principles of nutrition in growth and development of children may also be beneficial.

### 4.2 Limitations and Strong Points

The present study was, thus, beneficial in improving knowledge and attitude of the mothers. However knowledge doesn’t guarantee the proper action. Therefore we suggest that the mother’s performance, it’s relation to the consequences of diarrhea and nutritional status of the children might be considered in the future studies.
